# Coherence and Entanglement Dynamics in Training Variational Quantum Perceptron

**DOI:** 10.3390/e22111277

**Published:** 2020-11-11

**Authors:** Min Namkung, Younghun Kwon

**Affiliations:** 1 Departments of Applied Mathematics, Tikhonov Moscow Institute of Electronics and Mathematics, National Research University Higher School of Economics, 101000 Moscow, Russia; mslab.nk@gmail.com; 2 Departments of Applied Physics, Hanyang University, Ansan, Kyunggi-Do 425-791, Korea

**Keywords:** coherence, coherence distribution, coherence depletion, entanglement, quantum machine learning, quantum computer, quantum supremacy

## Abstract

In quantum computation, what contributes supremacy of quantum computation? One of the candidates is known to be a quantum coherence because it is a resource used in the various quantum algorithms. We reveal that quantum coherence contributes to the training of variational quantum perceptron proposed by Y. Du et al., arXiv:1809.06056 (2018). In detail, we show that in the first part of the training of the variational quantum perceptron, the quantum coherence of the total system is concentrated in the index register and in the second part, the Grover algorithm consumes the quantum coherence in the index register. This implies that the quantum coherence distribution and the quantum coherence depletion are required in the training of variational quantum perceptron. In addition, we investigate the behavior of entanglement during the training of variational quantum perceptron. We show that the bipartite concurrence between feature and index register decreases since Grover operation is only performed on the index register. Also, we reveal that the concurrence between the two qubits of index register increases as the variational quantum perceptron is trained.

## 1. Introduction

Quantum physics is known to provide better algorithms than its classical counterparts. Well-known examples include quantum superdense coding, quantum teleportation, and quantum key distribution [1]. When quantum physics is applied to computation, quantum computation algorithms can demonstrate remarkable improvements. In 1994, a factoring algorithm was suggested, that can factorize integer within a polynomial-time [2]. Furthermore, a quantum search algorithm was provided, which can effectively find a target in a large database [3,4,5,6,7]. For the implementation of quantum computation, many approaches have been proposed, such as linear optics [8,9], trapped ions [10], quantum dots [11], and superconductors [12]. Recently, artificial intelligence, in which quantum algorithms are applied, has been studied [13].

In quantum computation, what contributes supremacy of quantum computation? One of those candidates is entanglement [14,15]. D. Gottesman and I. L. Chuang suggested a universal quantum computer using quantum teleportation [16]. Because entanglement is a resource of quantum teleportation, Reference [16] reported that entanglement can contribute to a certain quantum computer.

However, entanglement is not a unique resource of quantum supremacy. This is supported by the argument of deterministic quantum computation with one-qubit (DQC1) [17]. Further, A. Datta et al. [18] insisted that quantum discord [19,20,21] can contribute to DQC1. It has been shown that quantum discord can be related not only to quantum computation [22,23], but also to quantum protocols such as quantum state merging [24], remote state preparation [25], and assisted optimal state discrimination [26,27,28,29].

Quantum correlations such as entanglement and quantum discord do not necessarily contribute to the performance of quantum computation. This can be supported by the Deutsch algorithm [30], where entanglement is not produced. When a maximally entangled state occurs in the algorithm, the Deutsch algorithm cannot be performed correctly [31]. Similar to the Deutsch algorithm, the success probability of the Grover algorithm is not relevant to entanglement, quantum discord, and non-locality [32].

In 2015, T. Baumgratz et al. proposed the quantity of *quantum coherence* [33]. Coherence can quantify the concept of superposition. Moreover, T. Baumgratz et al. suggested the mathematical conditions that the coherence measure should satisfy. Furthermore, they demonstrated that the $l_{1}-$norm coherence and relative entropy of coherence satisfied those conditions. Since the study of Reference [33], various coherence measures have been proposed [34,35].

The concept of coherence, which may explain wave-particle duality [36,37,38,39], may facilitate understanding of entanglement, quantum discord, and non-locality in a unified manner [40]. Therefore, coherence may contribute to various quantum algorithms. For example, J. Ma et al. [41] suggested that the quantum discord of DQC1 may be related to the consumption of coherence. Further, M. Hillery [42] insisted that coherence may contribute to performing Deutsch-Josza algorithm [43].

H.-L. Shi et al. [32] demonstrated that the success probability of the Grover algorithm and generalized Grover algorithm [44] was proportional to *coherence depletion*. Further, coherence depletion may be related to the performance of the Deutsch-Jozsa algorithm and Shor algorithm [45]. Therefore, coherence depletion can be considered as an important phenomenon in performing a quantum algorithm. Because the Grover algorithm can be used for the efficient implementation of quantum perceptron [46,47], coherence depletion may contribute to a machine learning task using a quantum property.

Recently, Y. Du et al. [47] proposed a variational quantum perceptron (VQP) using a multi-layer parametric quantum circuit (MPQC) and the Grover algorithm. In a VQP, the classical dataset is encoded on a quantum system composed of a feature register and an index register. After the MPQC and controlled-Z gate, the Grover algorithm is performed on the index register.

To understand the resource of the variational quantum perceptron, in this study, we investigate how coherence distribution [48,49,50] and coherence depletion occur in VQP training. First, we study the coherence distribution in the MPQC. We discover that when the VQP is correctly trained, the MPQC distributes the entire system’s coherence to the partial state in the index register. Further, as the number of iterations in training increases, the coherence of the partial state increases as well. In addition, we demonstrate that when VQP training is not correctly performed, the MPQC cannot distribute every coherence of the total system into the coherence of the partial state in the index register. This implies that accessible coherence disturbs VQP training, but the coherence of the partial state in the index register facilitates VQP training. Subsequently, we analyze the coherence depletion of the Grover algorithm. We discover that if VQP training is performed correctly, then coherence depletion occurs in the Grover algorithm. However, when VQP training is not performed correctly, coherence depletion does not occur in the Grover algorithm.

In addition, we analyze the behavior of entanglement in VQP training, by measuring concurrence [51,52,53]. First, we investigate the bipartite concurrence [51] between the feature and index registers after operating the MPQC. We discover that the bipartite concurrence between the feature and index registers diminishes. It is due to that Grover operation is only performed on the index register. Subsequently, we investigate the behavior of concurrence [52] of two qubits of index register after Grover operation. We show that the concurrence increases as VQP is successfully trained.

Our result can show how coherence and entanglement may influence the performance of the quantum computer and quantum perceptron. It tells that the quantum system’s various characters can affect the performance of the quantum computer and quantum perceptron. Further, our work can shed light on understanding quantum supremacy.

The remainder of this paper is organized as follows. In Section 2, we briefly explain the mathematical tool for coherence distribution. In Section 3, we present the behavior of coherence in VQP training. Specifically, we explain the distribution of coherence in the MPQC. Also, we investigate how coherence depletion occurs in the Grover algorithm. In Section 4, we discuss the coherence distribution and coherence depletion in two examples (Section 4.1 and Section 4.2). Also, we investigate the behavior of entanglement by evaluating concurrence in the two examples. In Section 5, we discuss and conclude our results.

## 2. Mathematical Tools for Coherence Distribution

In this section, we briefly describe the measures for evaluating coherence. The starting point is that the state without superposition [54] can be well expressed using a diagonalized operator having classical probability distributions [1]. Moreover, the state is defined as an incoherent state. Next, we denote the set of incoherent states as I. Subsequently, we can define a quantum operation, by which I is sent into I. The operation can be considered as an incoherent operation. If the operation preserves the trace, one can define the operation as an incoherent completely positive and trace-preserving map(ITPCPM). Hence, the conditions that the coherence measure C(·) should satisfy are expressed as follows:

(C1) The necessary and sufficient condition for C(δ)=0 is δ∈I.
(C2) Suppose that ICPTPM $\Phi \left(\cdot \right)=\sum_{i}K_{i}\left(\cdot \right)K_{i}^{†}$
(C2-1) ICPTPM does not increase coherence, which implies that C(ρ)≥C(Φ(ρ)).
(C2-2) A selective operation $K_{i}\left(\cdot \right)K_{i}^{†}$ does not increase coherence, which implies that $C\left(\rho \right)\geq \sum_{i}p_{i}C\left(\rho_{i}\right)$. Here, $p_{i}=\mathrm{Tr}\left(K_{i}\rho K_{i}^{†}\right)$, $\rho_{i}=K_{i}\rho K_{i}^{†}/p_{i}$.
(C3) State mixing does not increase coherence, which implies that the convexity $\sum_{i}p_{i}C\left(\rho_{i}\right)\geq C\left(\sum_{i}p_{i}\rho_{i}\right)$ holds.

One can see that the $l_{1}-$norm coherence ($C_{l_{1}}$) and the relative entropy of coherence ($C_{rel}$) satisfy the conditions above [33]. The $l_{1}-$norm coherence ($C_{l_{1}}$) and the relative entropy of coherence ($C_{rel}$) can be analytically defined as:

$$\begin{array}{ccc} C_{l_{1}}\left(\rho \right) & = & \min_{\delta \in I}||\rho -{\delta ||}_{l_{1}}=\sum_{i\neq j}\left|\rho_{ij}\right|,\\ C_{rel}\left(\rho \right) & = & \min_{\delta \in I}S\left(\rho ||\delta \right)=S\left(\delta \right)-S\left(\rho \right). \end{array}$$

Here, ${\left||M|\right|}_{l_{1}}=\sum_{i,j}\left|M_{ij}\right|$ is the $l_{1}-$norm, S(·||·) is the relative von Neumann entropy, and S(·) is the von Neumann entropy. Because $l_{1}-$norm coherence and relative entropy of coherence satisfy (C1), the minimum of these coherence measures is zero. Meanwhile, when $\rho_{d}$ is a *d*-dimensional maximally coherent state, the maximum of $l_{1}-$norm coherence (relative entropy of coherence) is d−1 ($\log_{2}d$).

Unlike a quantum correlation, coherence can be applied to a single system. In addition, the coherence of a multipartite system can be defined. For a bipartite system AB, the coherence $C(\rho_{AB})$ of $\rho_{AB}$ is expressed as [48]

$$\begin{array}{c} C\left(\rho_{AB}\right)=C\left(\rho_{A}\right)+C_{A}^{acc}+C\left(\rho_{B}\right)+C_{B}^{acc}+C_{AB}^{r}. \end{array}$$

Here, $\rho_{A}={\mathrm{Tr}}_{B}\rho_{AB}$ and $\rho_{B}={\mathrm{Tr}}_{A}\rho_{AB}$. $C_{A}^{acc}$($C_{B}^{acc}$) is the accessible coherence of system A(system B), where $C_{A}^{acc}$ is defined as follows:

$$\begin{array}{c} C_{A}^{acc}=\sum_{i}p_{i}C\left(\rho_{A,i}\right)-C\left(\rho_{A}\right),\;\;\rho_{A,i}=\frac{{\mathrm{Tr}}_{B}\left[I⊗\Pi_{i}\rho_{AB}I⊗\Pi_{i}\right]}{\mathrm{Tr}[I⊗\Pi_{i}\rho_{AB}I⊗\Pi_{i}]},\;\;p_{i}=\mathrm{Tr}\left[I⊗\Pi_{i}\rho_{AB}I⊗\Pi_{i}\right]. \end{array}$$

Here, $\Pi_{i}=|i\rangle \langle i|$ is an orthogonal basis of Hilbert space of system *B*. $C_{B}^{acc}$ can be defined similarly as $C_{A}^{acc}$. Therefore, the local coherence of system *A*(*B*) is given as $C_{A}=C\left(\rho_{A}\right)+C_{A}^{acc}$($C_{B}=C\left(\rho_{B}\right)+C_{B}^{acc}$). $C_{AB}^{r}$ is remaining coherence. The remaining coherence is not localized on system *A* or system *B*. If the $l_{1}-$norm coherence or the relative entropy of coherence is used as a coherence measure, then the remaining coherence becomes non-negative [48].

## 3. Coherence Processing in VQP Training

VQP is an quantum computer algorithm of a perceptron [55]. In a classical algorithm, a perceptron can be performed as follows. Suppose that a training dataset $D={\left\{\left({\vec{x}}_{i},y_{i}\right)\right\}}_{i=0}^{N-1}$ is provided. Here, ${\vec{x}}_{i}\in R^{M}$ is a data vector and $y_{i}\in \left\{+1,-1\right\}$ is the label corresponding to data vector ${\vec{x}}_{i}$. The purpose of perceptron is to find the hyperplane $\vec{W}\in R^{M}$ that minimizes $-\sum_{i=1}^{N}\mathrm{sign}\left(y_{i}\vec{W}\cdot {\vec{x}}_{i}\right)$. When in the VQP algorithm hyperplane $\vec{W}$ is correctly found, two halfspaces contain data vectors with correct labels. If a mislabeled data vector ${\vec{x}}_{k}$ is in the halfspace, we must find the ${\vec{x}}_{k}$ among every data vector in the half-space. When we use the Grover algorithm in this process, we can find the mislabeled data vector, with query complexity of $O(\sqrt{N})$.

**Figure 1:**
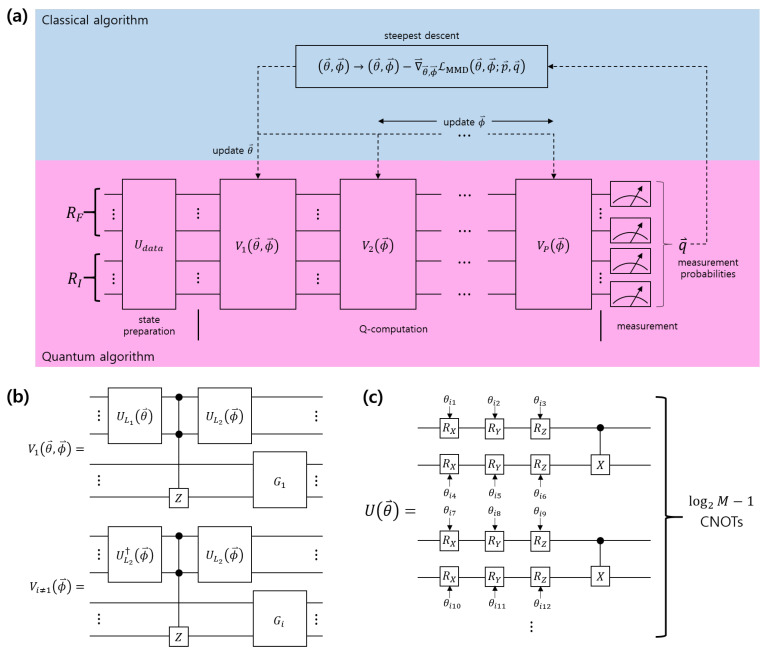
Schematic of variational quantum Perceptron (VQP) model [47]. (**a**) Entire VQP schematic is shown. (**b**) Structure of multi-layer parametric quantum circuit (MPQC) composing VQP. (**c**) Structure of quantum circuit $U(\vec{\theta })$ is shown.

The VQP proposed by Reference [47] is shown in Figure 1. It comprises a $\log_{2}M$ qubit feature register($R_{F}$) and $\log_{2}N$ qubit index register($R_{I}$). The initial state of the feature register and the index register is expressed as ${|0\rangle }^{⊗\log_{2}N+\log_{2}M}$. $U_{data}$ encodes the values of the dataset into the initial state. Suppose that the label of the *k*-th data vector is incorrect and $U_{data}$ provides a quantum state of $|\Phi_{k}\rangle \;=\;U_{data}{|0\rangle }^{⊗\log_{2}N+\log_{2}M}$. Hence, the quantum computer performs *P*-times of the multi-layer parametric quantum circuit (MPQC) $V_{1}\left(\vec{\theta },\vec{\phi }\right),V_{2}\left(\vec{\phi }\right),\cdots ,V_{P}\left(\vec{\phi }\right)$ on $|\Phi_{k}\rangle$. Here, $V_{1}\left(\vec{\theta },\vec{\phi }\right)$($V_{i\neq 1}\left(\vec{\phi }\right)$) depends on $\vec{\theta },\vec{\phi }$($\vec{\phi }$). $V_{i}$ is described in Figure 1b. In Figure 1b, $U_{L_{1}}\left(\vec{\theta }\right)$, $U_{L_{2}}\left(\vec{\phi }\right)$, $\vec{\theta }$, and $\vec{\phi }$ are expressed as $U_{L_{1}}\left(\vec{\theta }\right)=\prod_{i=1}^{L_{1}}U\left({\vec{\theta }}_{i}\right)$, $U_{L_{2}}\left(\vec{\phi }\right)=\prod_{i=1}^{L_{2}}U\left({\vec{\phi }}_{i}\right)$, $\vec{\theta }=\left({\vec{\theta }}_{1},\cdots ,{\vec{\theta }}_{L_{1}}\right)$, and $\vec{\phi }=\left({\vec{\phi }}_{1},\cdots ,{\vec{\phi }}_{L_{2}}\right)$, respectively. In $V_{1}\left(\vec{\theta },\vec{\phi }\right)$, $U_{L_{1}}\left(\vec{\theta }\right)$ and the multiqubit controlled-Z gate changes the phase of the mislabeled data. $U_{L_{2}}\left(\vec{\phi }\right)$ eliminates entanglement between the feature register and the index register. $G_{1}$ performs the Grover algorithm on the index register. In $V_{i\neq 1}\left(\vec{\phi }\right)$, $U_{L_{1}}$ is replaced by $U_{L_{2}}^{†}$. To perform an identical calculation, the entanglement between the feature register and the index register should be created. The quantum circuit $U({\vec{\theta }}_{i})$ can be found in Figure 1c. Here, $R_{X},R_{Y}$, and $R_{Z}$ are single-qubit rotation gates of the x,y, and *z* components, respectively.

When quantum computations are performed in *P*-times, quantum computer measures each qubit using a projective measurement ${\left\{|0\rangle \langle 0|,|1\rangle \langle 1|\right\}}^{⊗\log_{2}N}$. Suppose that the result of the measurement is $\vec{q}\left(\vec{\theta },\vec{\phi }\right)=\left(q\left(0;\vec{\theta },\vec{\phi }\right),\cdots ,q\left(N-1;\vec{\theta },\vec{\phi }\right)\right)$. Here, $q(i;\vec{\theta },\vec{\phi })$ is the probability that the result of the index register is *i*. The purpose of the VQP is to train the quantum circuit such that the measurement probability distribution $\vec{q}$ becomes close to the target probability distribution $\vec{p}=\left(p\left(0\right),\cdots ,p\left(N-1\right)\right)$. Here, when i≠k, we have p(i)=0. When i=k, we have p(k)=1. The training of a quantum circuit is provided by a classical algorithm. The measurement probability distribution, target probability distribution, and (untrained) circuit parameter determine the loss of the maximum mean discrepancy (MMD):

$$\begin{array}{c} L_{\mathrm{MMD}}\left(\vec{\theta },\vec{\phi };\vec{p},\vec{q}\right)=\sum_{x,y}q\left(x;\vec{\theta },\vec{\phi }\right)q\left(y;\vec{\theta },\vec{\phi }\right)K\left(x,y\right)-2\sum_{x,y}q\left(x;\vec{\theta },\vec{\phi }\right)p\left(y\right)K\left(x,y\right)+\sum_{x,y}p\left(x\right)p\left(y\right)K\left(x,y\right). \end{array}$$

Here, $K\left(x,y\right)=\exp(-|x-y{|}^{2}/2\sigma_{i})$ is a Gaussian kernel [56], and $\sigma_{i}$ is the bandwidth. The quantum circuit is trained in the manner in which the MMD loss diminishes. Assuming that the learning rate is *r*, the parameter is updated as $\left(\vec{\theta },\vec{\phi }\right)\to \left(\vec{\theta },\vec{\phi }\right)-r{\vec{\nabla }}_{\vec{\theta },\vec{\phi }}L_{\mathrm{MMD}}\left(\vec{\theta },\vec{\phi };\vec{p},\vec{q}\right)$ [57]. Here, ${\vec{\nabla }}_{\vec{\theta },\vec{\phi }}$ is the gradient with respect to $\vec{\theta }$ and $\vec{\phi }$.

The quantum algorithm comprises two processes. First, the MPQC should focus on the encoded information of the quantum state in the index register. Second, the Grover algorithm should effectively obtain the concentrated information in the index register. One can guess that the former is related to the coherence distribution, and the latter is related to coherence depletion.

### 3.1. Coherence Distribution Process

**Figure 2:**
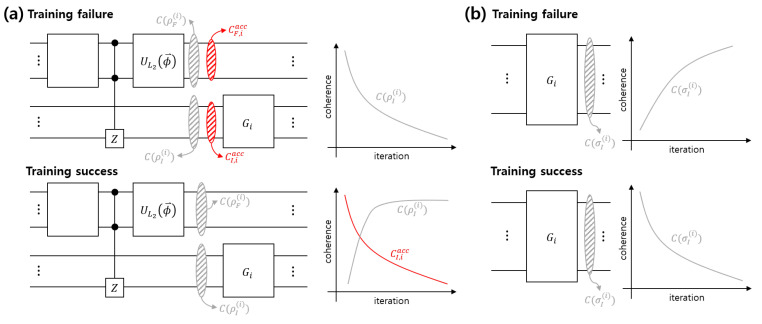
(**a**) Coherence distribution after operating *i*-th MPQC. In the schematic and graph, $C_{F,i}^{acc}$($C_{I,i}^{acc}$) is the accessible coherence of the feature register(the index register). (**b**) Coherence depletion after performing *i*-th Grover algorithm.

In the VQP, every information of dataset D is encoded into the feature and index registers. We can guess that for the Grover algorithm to find a mislabeled data, the MPQC should concentrate coherence into the index register. Therefore, we should train the VQP such that after operating the MPQC, the coherence distribution behaves as in the case of a successful training, as shown in Figure 2a. One should note that every coherence of the index register does not facilitate the Grover algorithm. The index register’s local coherence comprises the coherence and the accessible coherence of the partial state. We can see that the index register’s local coherence seems to be directly related to VQP training. The following two findings are obtained from our results. First, when the VQP is correctly trained, the accessible coherence of the index register and the feature register disappear. Second, the coherence of the index register state increases up to a specific value. Therefore, one can conclude that the accessible coherence hinders VQP training, whereas the coherence of the index register state facilitates VQP training.

### 3.2. Coherence Depletion Process

As we explained previously, the MPQC composing the VQP strengthens the coherence of the index register state. As shown in Figure 2b, when VQP training is correctly trained, the Grover algorithm consumes the coherence of the index register state. Further, we will demonstrate that even when during performing the Grover algorithm accessible coherence occurs, the Grover algorithm consumes the accessible coherence. However, as shown in Figure 2b, if VQP training is not correctly trained, the index register’s local coherence increases, which degrades the Grover algorithm’s performance. This is because coherence depletion should occur when the Grover algorithm is correctly performed [32].

## 4. Simulation Examples of Training

In this section, we analyze the relationship between VQP training and coherence, by using the examples in Reference [47].

### 4.1. Example 1

**Figure 3:**
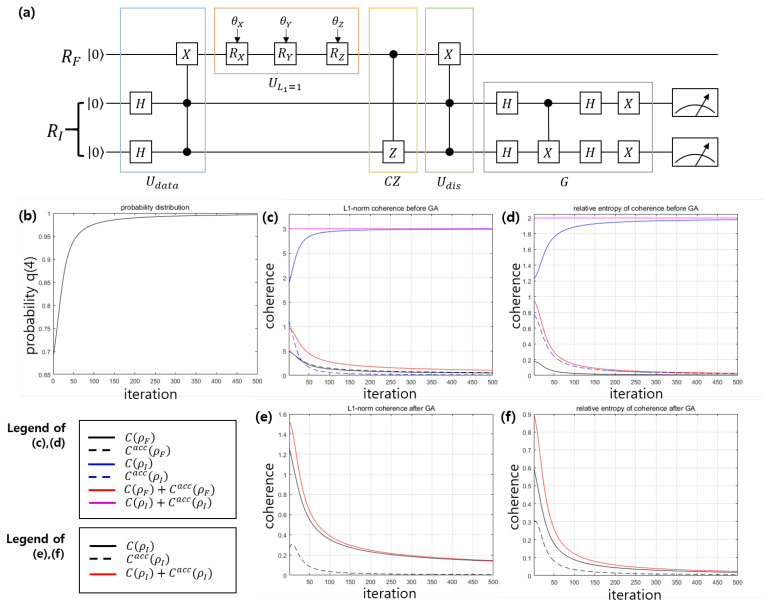
(**a**) Schematic of VQP model for dataset of Example 1. (**b**) Probability q(4) for the measurement result to be i=4. (**c**) $l_{1}-$norm coherence before performing Grover algorithm *G*. (**d**) Relative entropy of coherence before performing Grover algorithm *G*. (**e**) $l_{1}-$norm coherence after performing Grover algorithm *G*. (**f**) Relative entropy of coherence after performing Grover algorithm *G*. [Line description of (**c**,**d**)] Solid black (blue) line corresponds to a coherence of feature (index) register state $C(\rho_{F})$ ($C(\rho_{I})$). Dashed black (blue) line corresponds to an accessible coherence of feature (index) register $C^{acc}\left(\rho_{F}\right)$ ($C^{acc}\left(\rho_{I}\right)$). Solid red (purple) line corresponds to a local coherence of feature (index) register $C\left(\rho_{F}\right)+C^{acc}\left(\rho_{F}\right)$ ($C\left(\rho_{I}\right)+C^{acc}\left(\rho_{I}\right)$). [Line description of (**e**,**f**)] Solid black line corresponds to a coherence of index register state $C(\rho_{I})$. Dashed black line corresponds to an accessible coherence of index register $C^{acc}\left(\rho_{I}\right)$. Solid red line corresponds to a local coherence of index register $C\left(\rho_{I}\right)+C^{acc}\left(\rho_{I}\right)$.

We consider an example where the dataset is D={(1,0,1),(1,0,1),(1,0,1),(0,1,−1)} [47]. We assume that the mislabeled data is ${\vec{x}}_{4}=\left(0,1\right)$ in the dataset. The VQP for this problem is described in Figure 3a. In Figure 3a, $U_{data}$ is composed of the Hadamard gate and controlled-controlled-X gate. Because in this example we have M=2 and N=4, the MPQC comprise single qubit rotation gates $R_{X},R_{Y}$, and $R_{Z}$. The disentanglement gate can be constructed by the controlled-controlled-X gate. If training can be performed correctly, the measurement probability $\vec{q}\left(\vec{\theta }\right)$ converges to the target probability $\vec{p}=\left(0,0,0,1\right)$.

In the process of training VQP of Figure 3a, the coherence distribution and the coherence depletion are displayed in Figure 3b–f. Figure 3c,e show the $l_{1}-$norm coherence, and Figure 3d,f show the relative entropy of coherence. Since the index register (feature register) is a 4-dimensional (2-dimensional) quantum system, the maximum of $l_{1}-$norm coherence is 3 (2), and the maximum of relative entropy of coherence is 2 (1). As shown in Figure 3, the local coherence of the index register(purple solid line) shows maximal coherence regardless of the iteration. Meanwhile, the accessible coherence of the index register(dotted blue line) converges to zero as the number of iterations increases. In addition, the coherence of the index register state(solid blue line) converges to local coherence. This implies that the coherence of the index register state contributes to the VQP training.

As shown in Figure 3, the coherence of the feature register state(solid black line) converges to zero. Also, the accessible coherence of the feature register(dotted black line) converges to zero. Therefore, the local coherence of the feature register decreases as the number of iterations increases.

### 4.2. Example 2

This example has the dataset $D={\left\{\left({\vec{x}}_{i},y_{i}\right)\right\}}_{i=0}^{7}$, given by the following data vectors [47]:

$$\begin{array}{ccc} {\vec{x}}_{0} & = & \left[0.6,0.8,-\frac{1}{\sqrt{2}},-\frac{1}{\sqrt{2}}\right],\;\;{\vec{x}}_{1}=\left[1,0,-\frac{1}{\sqrt{3}},-\sqrt{\frac{2}{3}}\right],\;\;{\vec{x}}_{2}=\left[0,1,-\frac{1}{3},-\frac{\sqrt{8}}{3}\right],{\vec{x}}_{3}=\left[\frac{1}{2},\frac{\sqrt{3}}{2},-1,0\right],\\ {\vec{x}}_{4} & = & \left[0.8,0.6,-\frac{\sqrt{3}}{3},-\frac{\sqrt{6}}{3}\right],\;\;{\vec{x}}_{5}=\left[\sqrt{\frac{6}{7}},\frac{1}{\sqrt{7}},-\frac{1}{\sqrt{5}},-\frac{\sqrt{20}}{5}\right],\;\;{\vec{x}}_{6}=\left[-\frac{\sqrt{3}}{3},-\frac{\sqrt{3}}{6},0.6,0.8\right],\\ {\vec{x}}_{7} & = & [-\frac{\sqrt{3}}{3},-\frac{\sqrt{3}}{6},0.6,0.8]. \end{array}$$

**Figure 4:**
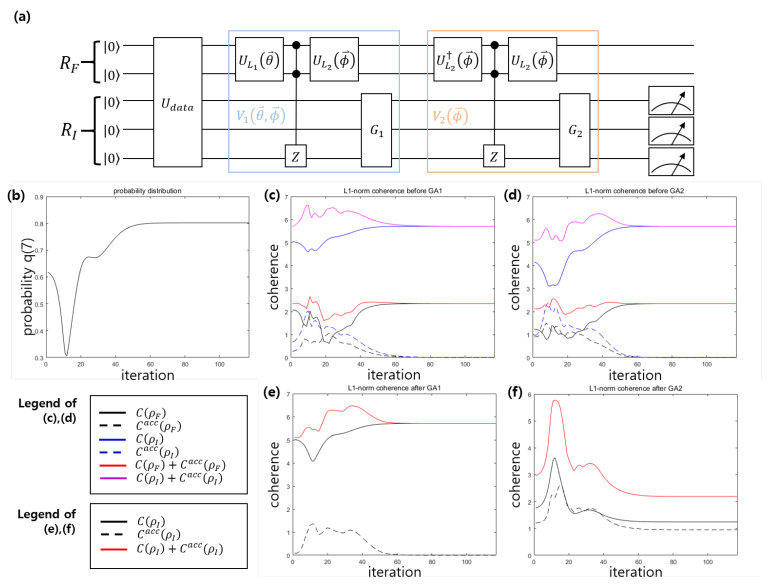
VQP model of example 2 [47] for succeful case. (**a**) The schematic of VQP model. (**b**) The success probability of VQP. (**c**) $l_{1}-$norm coherence before performing GA1. (**d**) $l_{1}-$norm coherence before performing GA2. (**e**) $l_{1}-$norm coherence after performing GA1. (**f**) $l_{1}-$norm coherence after performing GA2. [Line description of (**c**,**d**)] Solid black (blue) line corresponds to the coherence of feature (index) register state $C(\rho_{F})$ ($C(\rho_{I})$). Dashed black (blue) line corresponds to the accessible coherence of feature (index) register $C^{acc}\left(\rho_{F}\right)$ ($C^{acc}\left(\rho_{I}\right)$). Solid red (purple) line corresponds to the local coherence of feature (index) register $C\left(\rho_{F}\right)+C^{acc}\left(\rho_{F}\right)$ ($C\left(\rho_{I}\right)+C^{acc}\left(\rho_{I}\right)$). [Line description of (**e**,**f**)] Solid black line corresponds to the coherence of index register state $C(\rho_{I})$. Dashed black line corresponds to the accessible coherence of index register $C^{acc}\left(\rho_{I}\right)$. Solid red line corresponds to the local coherence of index register $C\left(\rho_{I}\right)+C^{acc}\left(\rho_{I}\right)$.

Here, the correct classification is defined as follows: For data vector ${\vec{x}}_{i}=\left[x_{i1},x_{i2},x_{i3},x_{i4}\right]$, when $x_{i1},x_{i2}>0$ and $x_{i3},x_{i4}\leq 0$, the label $y_{i}=+1$ is assigned, but when $x_{i1},x_{i2}\leq 0$ and $x_{i3},x_{i4}>0$, the label $y_{i}=-1$ is assigned [47]. In this example, we assume that $y_{0}=y_{1}=\cdots =y_{5}\;=\;+1,y_{6}\;=\;-1,y_{7}=1$, which means that the mislabeled data is $y_{7}$.The VQP model for this example is shown in Figure 4. Because the number of mislabeled data is one, the number of optimal iterations is l=(π−2θ)/4θ≃1.6734 [3]. Here, we have $\theta =\sin^{-1}\left(1/\sqrt{8}\right)$. Therefore, the VQP model contains two Grover operations $G_{1}$ and $G_{2}$. In addition, we have $L_{1}=L_{2}=3$.

After training is completed, the probability of success becomes 80.2%. When the number of iterations is 12, the success probability of the VQP becomes a local minimum. Since the index register (feature register) is an 8-dimensional (4-dimensional) quantum system, the maximum of $l_{1}-$norm coherence is 7 (3), and the maximum of relative entropy of coherence is 3 (2). Figure 4c–f show the $l_{1}-$norm coherence before(after) performing $G_{1}$ and $G_{2}$. The success probability in Figure 4b is similar to the coherence of the index register state shown in Figure 4c,d. When the number of iterations is approximately 12, the coherence of the index register state(solid blue line) becomes a minimum. As the number of iterations increases, the coherence of the index register state converges to a maximum. Meanwhile, the accessible coherence of the index register state(dotted blue line) and the feature register state(dotted black line) vanishes as the number of iterations increases. Therefore, most of the local coherence in the index register is the coherence of the index register state. Further, the manner in which $G_{1}$ and $G_{2}$ affect the local coherence of the index register is noteworthy. That is, while $G_{1}$ does not consume the local coherence, the local coherence of the index register diminishes in $G_{2}$. Therefore, to understand VQP training, it is important to determine where coherence depletion occurs in the Grover operation.

**Figure 5:**
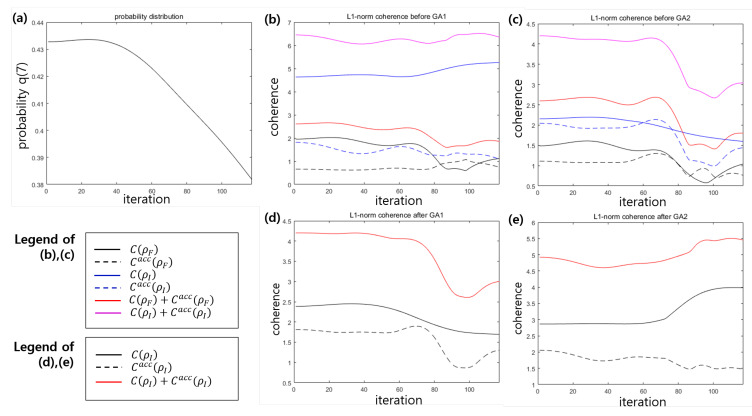
VQP model of example 2 [47] for a failed case. (**a**) The success probability of VQP. (**b**) $l_{1}-$norm coherence before performing GA1. (**c**) $l_{1}-$norm coherence before performing GA2. (**d**) $l_{1}-$norm coherence after performing GA1. (**e**) $l_{1}-$norm coherence after performing GA2. [Line description of (**b**,**c**)] Solid black (blue) line corresponds to the coherence of feature (index) register state $C(\rho_{F})$ ($C(\rho_{I})$). Dashed black (blue) line corresponds to the accessible coherence of feature (index) register $C^{acc}\left(\rho_{F}\right)$ ($C^{acc}\left(\rho_{I}\right)$). Solid red (purple) line corresponds to the local coherence of feature (index) register $C\left(\rho_{F}\right)+C^{acc}\left(\rho_{F}\right)$ ($C\left(\rho_{I}\right)+C^{acc}\left(\rho_{I}\right)$). [Line description of (**d**,**e**)] Solid black line corresponds to the coherence of index register state $C(\rho_{I})$. Dashed black line corresponds to the accessible coherence of index register $C^{acc}\left(\rho_{I}\right)$. Solid red line corresponds to the local coherence of index register $C\left(\rho_{I}\right)\;+\;C^{acc}\left(\rho_{I}\right)$.

Figure 5 shows the case where VQP training fails. Here, we consider $y_{0}=y_{1}=\cdots =y_{5}=y_{6}=+1,y_{7}=-1$, where the mislabeled data is $y_{7}$. As shown in Figure 5a, the success probability q(7) decreases from 43.28% to 38.26%. In Figure 5a,b, the accessible coherence of the index register (dotted blue line) and the accessible coherence of the feature register (dotted black line) do not vanish. Further, the coherence of the index register state(blue solid line) diminishes constantly before performing $G_{2}$. Figure 5c,d shows that the coherence depletion occurs in $G_{1}$, but the coherence increase in $G_{2}$. This implies that these Grover operations cannot consume the $l_{1}-$norm coherence appropriately.

### 4.3. Investigation of Entanglement in Two Examples

**Figure 6:**
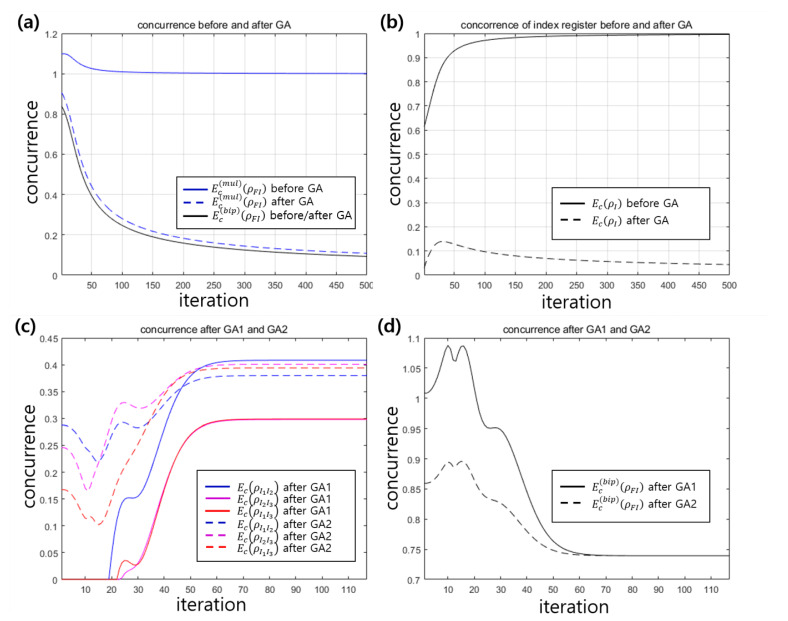
(**a**) Concurrence before and after Grover algorithm in Example 1. (**b**) Concurrence of index register before and after Grover algorithm in Example 1. (**c**) Concurrence of two qubit system $I_{i}I_{j}$ (i,j∈{1,2,3}) before and after GA1 and GA2 in Example 2. (**d**) Bipartite concurrence between feature and index register after GA1 and GA2 in Example 2. [Line description of (**a**)] Solid blue line corresponds to the multipartite concurrence $E_{c}^{\left(mul\right)}\left(\rho_{FI}\right)$ before GA. Dashed blue line corresponds to the multipartite concurrence $E_{c}^{\left(mul\right)}\left(\rho_{FI}\right)$ after GA. The solid black line is the bipartite concurrence $E_{c}^{\left(bip\right)}\left(\rho_{FI}\right)$ between the feature and index register before and after GA. [Line description of (**b**)] Solid black line corresponds to the concurrence of index register $E_{c}\left(\rho_{I}\right)$ before GA. Dashed black line corresponds to the concurrence of index register $E_{c}\left(\rho_{I}\right)$ after GA. [Line description of (**c**)] Solid blue, purple, and red lines correspond to the concurrence $E_{c}\left(\rho_{I_{1}I_{2}}\right)$, $E_{c}\left(\rho_{I_{2}I_{3}}\right)$, and $E_{c}\left(\rho_{I_{1}I_{3}}\right)$ after GA1, respectively. Dashed blue, purple, and red lines correspond to the concurrence $E_{c}\left(\rho_{I_{1}I_{2}}\right)$, $E_{c}\left(\rho_{I_{2}I_{3}}\right)$, and $E_{c}\left(\rho_{I_{1}I_{3}}\right)$ after GA2, respectively. [Line description of (**d**)] Solid black line corresponds to the bipartite concurrence between feature and index register $E_{c}^{\left(bip\right)}\left(\rho_{FI}\right)$ after GA1. Dashed black line corresponds to the bipartite concurrence between feature and index register $E_{c}^{\left(bip\right)}\left(\rho_{FI}\right)$ after GA2.

One of the known resources of quantum supremacy is entanglement. Therefore, in this study, we verify whether entanglement contributes to the performance of the VQP model. Figure 6 shows the concurrence [51,52,53] of two examples discussed in the previous section. The concurrence between two-qubit state $\rho_{I_{i}I_{j}}$ of index register $I_{i}$ and $I_{j}$ is defined as follows [52]:

$$\begin{array}{c} E_{c}\left(\rho_{I_{i}I_{j}}\right)=\max\left\{\sqrt{\lambda_{1}}-\sqrt{\lambda_{2}}-\sqrt{\lambda_{3}}-\sqrt{\lambda_{4}},0\right\}. \end{array}$$

Here, $\lambda_{i}$ is eigenvalue of $\rho_{I_{i}I_{j}}\sigma_{y}⊗\sigma_{y}\rho_{I_{i}I_{j}}^{*}\sigma_{y}⊗\sigma_{y}$, and the relation $\lambda_{1}>\lambda_{2}>\lambda_{3}>\lambda_{4}$ is required. Also the bipartite concurrence between the feature register and the index register is defined as [51]

$$\begin{array}{c} E_{c}^{\left(bip\right)}\left({|\psi \rangle }_{FI}\right)=\sqrt{2(1-\mathrm{Tr}\rho_{I}^{2})}. \end{array}$$

In Example 1, the multipartite entanglement of the entire system is defined as follows [53]:

$$\begin{array}{ccc} & & E_{c}^{\left(mul\right)}\left({|\psi \rangle }_{FI}\right)=\frac{2}{\sqrt{8}}\sqrt{6-\sum_{\alpha =1}^{6}\rho_{\alpha }^{2}},\\ & & \rho_{1}=\rho_{F},\;\;\rho_{2}=\rho_{I_{1}},\;\;\rho_{3}=\rho_{I_{2}},\;\;\rho_{4}=\rho_{FI_{1}},\;\;\rho_{5}=\rho_{FI_{2}},\;\;\rho_{6}=\rho_{I_{1}I_{2}}. \end{array}$$

Because the quantum state of the entire system is a pure state, $E_{c}^{\left(bip\right)}$ and $E_{c}^{\left(mul\right)}$ can be descried as above. The maximum of concurrence of two-qubit state is one, but the maximum of bipartite concurrence is given by $\sqrt{2\left(d_{F}-1\right)/d_{F}}$, (Here is the proof. Suppose that the dimension $d_{F}$ of the feature register is smaller than the dimension $d_{I}$ of the index register. Then, the pure entangled state of these register can be described as ${|\psi \rangle }_{FI}=\sum_{i=1}^{d_{F}}\lambda_{i}{|\lambda_{i}\rangle }_{F}⊗{|\lambda_{i}\rangle }_{I}$, from Schmidt decomposition. Here, $\left\{{|\lambda_{i}\rangle }_{X}\right\}$ is an orthonormal basis of system X∈{F,I}. In the case of maximal entanglement, we can have $\lambda_{i}=1/\sqrt{d}$ (∀i). Then, the trace of the square of the partial state of |ψ〉〈ψ| becomes $1/d_{F}$. Substituting the value, we find that the maximum of bipartite concurrence is given by $\sqrt{2\left(d_{F}-1\right)/d_{F}}$.) where $d_{F}$ is the dimension of the feature register. In Example 1, the maximum of bipartite concurrence is one because of $d_{F}=2$. However, in Example 2, the maximum of bipartite concurrence is given as $\sqrt{3/2}≃1.2247$, (For example, in Figure 6d, when the iteration is 10, the bipartite concurrence becomes 1.088. It implies that when the iteration is 10, the state between the feature register and the index register is close to the maximally entangled state.) because of $d_{F}=4$. Here, Figure 6a,b is the case of success in example 1, and Figure 6c,d is the case of success in example 2. In Figure 6a, the solid blue line(the dashed line) is the multipartite concurrence before(after) the Grover operation is performed [53]. Further, we consider the bipartite concurrence between the feature register and the index register. The solid(dashed) black line is the bipartite concurrence between the feature register and the index register before(after) Grover operation is performed. Figure 6a shows that by performing the Grover operation the multipartite concurrence converges to the bipartite concurrence, and by iterating Grover operation the bipartite concurrence disappears. This implies that entanglement should diminish for the successful training of the VQP model. Figure 6b shows the concurrence [52] of qubits composing the index register. When VQP training is performed correctly, the concurrence before the Grover operation is performed converges to the value corresponding to the maximally entangled state. However, after the Grover operation, the concurrence disappears. This coincides with previous results [32] which implied that entanglement should be removed for the Grover algorithm to be performed correctly. Figure 6c,d show that the bipartite concurrence between the index register and the feature register diminishes. It is noteworthy that unlike those shown in Figure 6a,b, the bipartite concurrence does not converge to zero. Also, the success probability of the VQP is less than one owing to non-zero concurrence. Figure 6c shows that $G_{2}$ increases the value of the concurrence among the two qubits of the index register as the VQP is successfully trained. Figure 6d shows that $G_{2}$ decreases the bipartite concurrence between the feature and the index register. The phenomena can be understood because the Grover algorithm is only operated in the index register.

## 5. Conclusions

In this study, we investigated the contribution of coherence to the training of the VQP model. First, we discovered that before performing MPQC, coherence should be concentrated on the index register. Second, we demonstrated that the Grover algorithm consumes the coherence of the index register. We discovered that coherence distribution and coherence depletion occur in correctly trained VQP model. Further, we demonstrated that to train the VQP model correctly, the local coherence of the index register should not contain accessible coherence [48]. Also, we investigated whether entanglement may affect to training the VQP model. We discovered that VQP model should be trained not to produce entanglement. Finally, we demonstrated that entanglement depletion does not occur in VQP training. Our result provided how coherence and entanglement may influence the performance of the quantum computer and quantum perceptron. And our work will help to understand the essence of quantum supremacy.

## Abbreviations

The following abbreviations are used in this manuscript:

ITPCPM	Incoherent completely positive and trace-preserving map
DQC1	Deterministic quantum computation with one-qubit
VQP	Variational quantum Perceptron
MPQC	Multi-layer parametric quantum circuit
